# Prospective study on the mismatch concept in acute stroke patients within the first 24 h after symptom onset - 1000Plus study

**DOI:** 10.1186/1471-2377-9-60

**Published:** 2009-12-08

**Authors:** Benjamin Hotter, Sandra Pittl, Martin Ebinger, Gabriele Oepen, Kati Jegzentis, Kohsuke Kudo, Michal Rozanski, Wolf U Schmidt, Peter Brunecker, Chao Xu, Peter Martus, Matthias Endres, Gerhard J Jungehülsing, Arno Villringer, Jochen B Fiebach

**Affiliations:** 1 Center for Stroke Research Berlin, Charité - Universitätsmedizin Berlin, Charitéplatz 1, 10117 Berlin, Germany; 2 Advanced Medical Research Center, Iwate Medical University, 19-1 Uchimaru, Morioka 020-8505, Japan; 3 Institute of Biometrics and Clinical Epidemiology, Charité - Universitätsmedizin Berlin, Charitéplatz 1, 10117 Berlin, Germany

## Abstract

**Background:**

The mismatch between diffusion weighted imaging (DWI) lesion and perfusion imaging (PI) deficit volumes has been used as a surrogate of ischemic penumbra. This pathophysiology-orientated patient selection criterion for acute stroke treatment may have the potential to replace a fixed time window. Two recent trials - DEFUSE and EPITHET - investigated the mismatch concept in a multicenter prospective approach. Both studies randomized highly selected patients (n = 74/n = 100) and therefore confirmation in a large consecutive cohort is desirable. We here present a single-center approach with a 3T MR tomograph next door to the stroke unit, serving as a bridge from the ER to the stroke unit to screen all TIA and stroke patients. Our primary hypothesis is that the prognostic value of the mismatch concept is depending on the vessel status. Primary endpoint of the study is infarct growth determined by imaging, secondary endpoints are neurological deficit on day 5-7 and functional outcome after 3 months.

**Methods and design:**

1000Plus is a prospective, single centre observational study with 1200 patients to be recruited. All patients admitted to the ER with the clinical diagnosis of an acute cerebrovascular event within 24 hours after symptom onset are screened. Examinations are performed on day 1, 2 and 5-7 with neurological examination including National Institute of Health Stroke Scale (NIHSS) scoring and stroke MRI including T2*, DWI, TOF-MRA, FLAIR and PI. PI is conducted as dynamic susceptibility-enhanced contrast imaging with a fixed dosage of 5 ml 1 M Gadobutrol. For post-processing of PI, mean transit time (MTT) parametric images are determined by deconvolution of the arterial input function (AIF) which is automatically identified. Lesion volumes and mismatch are measured and calculated by using the perfusion mismatch analyzer (PMA) software from ASIST-Japan. Primary endpoint is the change of infarct size between baseline examination and day 5-7 follow up.

**Discussions:**

The aim of this study is to describe the incidence of mismatch and the predictive value of PI for final lesion size and functional outcome depending on delay of imaging and vascular recanalization. It is crucial to standardize PI for future randomized clinical trials as for individual therapeutic decisions and we expect to contribute to this challenging task.

**Trial Registration:**

clinicaltrials.gov NCT00715533

## Background

Imaging is a central element of stroke diagnostics in clinical routine and randomized clinical trials. With respect to the label of alteplase, the only approved drug for acute ischemic stroke treatment, many clinicians consider CT the gold standard for stroke imaging during the first 3 hours of ictus. CT is implemented in the clinical workflow at many centers and allows ruling out hemorrhage easily. However, its sensitivity for ischemic infarction is 40% with a negative predictive value of 17% [1] and hence the accuracy to prevent stroke mimic patients from alteplase treatment is low. Over the last couple of years it has been shown, that multiparametric MRI is as sensitive to intracerebral hemorrhage (ICH) as CT in an acute stroke setting [2,3]. In addition MRI offers complex information about infarction and perfusion deficits, vessel status and extent of subacute and chronic lesions, within an examination lasting less than 15 minutes. Several open case series and multicenter studies underlined the usefulness of MRI in an acute setting for therapeutic decisions [4-6]. The mismatch between diffusion restriction and perfusion deficit volumes has been interpreted as the surrogate of ischemic penumbra, showing the tissue at risk of infarction - the target tissue for therapeutic intervention [7-9]. MRI has been used to replace a narrow time window for thrombolysis by a pathophysiology orientated patient selection [10,11].

Recently several studies and interventional trials have used MRI to assess stroke patients. Parsons et al. demonstrated the feasibility of expanding the thrombolysis time window by selecting eligible patients via MRI in an open case series. The trials investigating the safety and efficacy of desmoteplase selected patients by MR imaging [7,8]. Only patients presenting with a diffusion-weighted imaging (DWI) lesion volume less than a third of the middle cerebral artery (MCA) territory and presenting with an imaging surrogate of penumbra have been randomized. The 'Desmoteplase In Acute Ischemic Stroke' (DIAS) and 'Dose Escalation of Desmoteplase for Acute Ischemic Stroke' (DEDAS) trials show a significant correlation between early reperfusion at 6 hours and clinical outcome assessed on day 90. There are several possible explanations to the failing proof of the mismatch concept in the recently published DIAS II trial - a less severe baseline National Institute of Health Stroke Scale (NIHSS) score, missing vessel occlusion in a substantial percentage of cases and an unexpectedly high rate of spontaneous recoveries in the placebo group being the most important ones. Furthermore, Hill pointed out the dependence of the imaging hypothesis from a proven efficacy of the study drug [12,13].

Two investigator-initiated trials named 'Diffusion and Perfusion Imaging Evaluation For Understanding Stroke Evolution' (DEFUSE) and 'Echoplanar Imaging Thrombolytic Evaluation Trial' (EPITHET) investigated the advantages of MRI in acute stroke [14,15]. Both trials used alteplase to treat patients and were limited by low recruiting rates. DEFUSE screened 1020 patients for a total enrolment of 74 patients over a period of four years. A secondary analysis of the DEFUSE population with technically adequate MR angiographies (MRA) suggests that recanalization has a significant influence on the reliability of the mismatch concept [16]. EPITHET had a 7 years recruiting period. After screening 3908 patients 101 had been included. These data based on a selected population are not a sufficient basis to judge the mismatch concept for a broad population.

Having the opportunity of a 3T MR scanner next door to the stroke unit and general neurologic ward serving as a bridge from the emergency ward to a timely thrombolytic therapy we are enabled to image all stroke and TIA patients. We will measure the incidence of different imaging patterns and hypothesize that the prognostic value of mismatch differs based on MRA findings and delay between the time of onset and MRI examination.

## Methods and design

### Study Design

"1000Plus" is designed as a prospective, single centre observational study conducted by the Center for Stroke Research Berlin at the Campus Benjamin Franklin of the Charité University Hospital Berlin. The protocol received approval by the local Ethics Committee (EA4/026/08). MRI examination is routinely performed even if the patient is unable to give informed consent due to expected individual benefit and to avoid selection bias against aphasic patients. Study entry and storage of data require subsequent consent by the patient or a legally authorized representative.

### Duration and expected study completion

A screening period of 24 months is scheduled. End of study will be at the 3 month follow up of the last patient in. Recruiting has started 1-9-2008. Expected study completion date 1-12-2010. A further 6 months are planned for the evaluation of the predefined primary and secondary hypotheses.

### Patients

**Figure 1:**
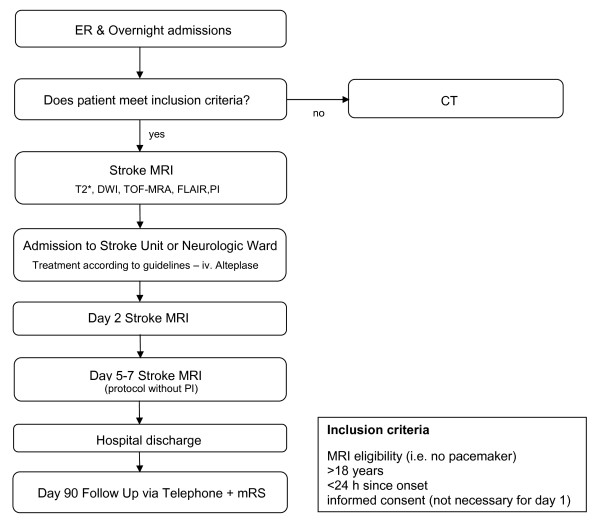
**Study Design**.

1200 Patients are going to be allocated to the trial. We estimated this inclusion goal on the basis of DEFUSE and EPITHET interpolated to our wider inclusion criteria towards TIAs and strokes with a minor deficit. With our given setting of about 1200 patients with an acute cerebrovascular event admitted to the emergency ward every year we expect to exam about 800 patients/year and about 600 of them are expected to give informed consent. An interims analysis of the first 3 months of inclusion is currently ongoing. A second analysis after the enrolment of 600 patients is planned. Those analyses will also be used to confirm the inclusion rates and power estimations. For a flow chart of study design and patient inclusion see Figure 1.

### Inclusion criteria

1. Patients admitted to the emergency ward with the clinical diagnosis of an acute cerebrovascular event within the last 24 hours who match the general MRI eligibility criteria.

2. Age older than 18 years

### Exclusion criteria

1. Pregnancy

2. Contraindications to MRI (i.e. pacemaker)

## Methods

### A. Stroke MRI

All examinations are performed with a 3T MR scanner (Tim Trio; Siemens AG, Erlangen, Germany) fully dedicated to clinical research. Our MRI protocol for acute stroke patients contains the following sequences for the day of admission (day 1) and day 2: T2*-weighted imaging to screen for ICH; DWI to assess cerebral infarction; time-of-flight MR-Angiography (TOF MRA) to detect vessel occlusion; Fluid-attenuated inverse recovery (FLAIR) to estimate microangiopathic lesions load and to investigate the age of the recent lesion; perfusion imaging (PI) to determine the tissue at risk. On day 5-7 a third measurement with a shorter protocol - without PI - will be performed to assess the final infarct size on FLAIR. PI is being conducted as dynamic susceptibility-enhanced contrast imaging. We apply a fixed dosage of 5 ml Gadovist^® ^(Gadobutrol, 1 M, Bayer Schering Pharma AG, Berlin, Germany) followed by 20 ml saline by a power injector (Spectris, Medrad Inc., Warrendale PA, USA) both at a rate of 5 ml/s. Patients weighing ≥100 kg or ≤50 kg receive 6 ml or 4 ml contrast agent respectively. For sequence parameters see Table 1.

**Table 1:** MRI protocol parameters in detail

	T2*WI	DWI	3D TOF-MRA	FLAIR	PWI
*TR, ms*	620.0	7600	22.0	8000	1390

*TE, ms*	20.0	93.0	3.86	100	29.0

*b, s/mm*^2^	-	0 (b0)	-	-	-
		1000 (b1)			

*Flip angle*	20°	-	18°	130°	60°

*Averages*	1	2	1	1	1 each 1.3-1.5 s

*FOV, mm*	220	230	200	220	230

*Matrix*	256 × 192	192 × 192	384 × 268	256 × 256	128 × 128

*Thickness, mm*	5.0	2.5	0.65	5.0	5

*Gap, mm*	0.5	0.0	-7,2	0.5	0.5

*Slices, n*	25	50		25	21 each 1.3-1.5 s

*Duration (mm:ss)*	01:13	02:11	03:50	01:54	01:58

For post-processing of PI, mean transit time (MTT) parametric images are determined by deconvolution of the arterial input function (AIF) for which multiple voxels are automatically identified. MTT maps are generated after sSVD (standard singular value decomposition) and bSVD (block-circulant SVD) deconvolution with the Perfusion Mismatch Analyzer software (PMA). Therefore, we will be able to compare a non-delay-corrected (sSVD) to a delay-corrected (bSVD) approach. If the interim analysis presents a significant superiority of one post-processing method over the other, all further data will be processed in that manner. Raters semi-automatically define a region of interest (ROI) in DWI, PI-maps and FLAIR images to measure infarct and perfusion deficit volumes. Volumes and mismatch are then calculated by using the PMA software from the Acute Stroke Imaging Standardization Group (ASIST) Japan (Version 3.1 or later).

### B. Scales

The NIHSS is a validated instrument used in many clinical trials to assess the neurologic deficit of stroke patients in a structured quick way. It has several items testing the subjects' consciousness, limb movement, sensorium, speech, vision and eye movements. NIHSS score is assessed on admission (day 1), day 2 and day 5-7 immediately before imaging. The modified Rankin Scale (mRS) is used to assess functional recovery in randomized stroke trials. In this study, mRS is rated 90 days after onset of symptoms to quantify handicap before and after the event. Demographic data and course of symptoms are documented according to the German Stroke database registry guidelines as well as stroke subtype and etiology. Delays between onset, arrival and imaging and duration of hospitalization are recorded.

### C. Outcome

The primary endpoint of 1000Plus is the change of infarct size between the baseline examination and the day 5-7 follow up. Baseline diffusion lesion and perfusion deficit volumes will be calculated by using the PMA software from ASIST-Japan. Comparison to the final lesion size on FLAIR at day 5-7 will be made. Secondary endpoints are clinical deficit on day 5-7 and functional recovery on day 90 according to NIHSS and mRS score respectively.

### D. Analysis

The mismatch between DWI and perfusion imaging is a surrogate of the ischemic penumbra. We hypothesize that the prognostic value differs depending on recanalization as shown by MRA on day 2. Furthermore we propose it declines with increasing delay between time of symptom onset and MR examination.

#### Dichotomization

We will dichotomize results based on whether there was an initial vessel occlusion or not and whether an initially obstructed vessel was recanalized at day 2 as shown on MRA. Comparison will be made between the groups regarding the predictive value of PI. We expect an accurate prediction of infarct size by DWI if a vessel occlusion is recanalized on day 2 and an accurate prediction by PI if the occlusion persists.

#### Time Stratification

We will group patients according to their delay between symptom onset and MR imaging time. Predefined time frames are ≤1 hour, 1-3 hours, 3-9 hours, 9-12 hours and 12-24 hours. We expect better positive and negative predictive values and accuracy the sooner a patient is examined.

#### CT control group

In order to evaluate whether there is selection bias in the study cohort, we will compare demographic values such as age and baseline stroke severity, etiology and subtype with all patients admitted to the hospital over the same time period that were not allocated to the study.

#### Stroke subtype and etiology

Mulivariate analyses will be used to look for changes in positive predictive values and accuracy of the model in different etiologies and subtypes.

## Discussion

The primary hypothesis of 1000Plus is that the prognostic value of PI is crucially depending on the vessel status. We expect DWI-PI-mismatch to be a more accurate parameter for treatment decisions if a vessel occlusion, the eventual target for thrombolysis, is apparent. We hypothesize that perfusion deficits in patients with patent vessels do not predict infarction.

### Selection

The aim of the study design was to include as many patients with cerebral ischemia as possible. Therefore we decided to keep inclusion and exclusion criteria as open as possible to avoid inclusion bias and production of unrepresentative data and chose to include transient ischemic attacks and strokes with a minor neurological deficit as well. The maximum delay between time of symptom onset and imaging was set to 24 hours since perfusion deficit declines as delay increases [17]. DEFUSE tested the hypothesis that mismatch patients have a better outcome after early reperfusion as opposed to mismatch patients without early reperfusion and non-mismatch patients. Only patients who could receive alteplase within 6 hours from stroke onset were included [14]. EPITHET tested the hypothesis that alteplase attenuates infarct growth in mismatch patients. Mismatch was not an inclusion criterion, whereas inclusion within 3-6 hours after onset was. EPITHET was underpowered to prove its primary hypothesis [15]. Our selection criteria accept patients with a smaller impairment and a longer time from onset to imaging but will still provide us with the possibility of comparison via stratification. Patients with a small impairment were previously underrepresented in studies investigating the mismatch model. Especially those patients could benefit from mismatch-guided thrombolysis and avoidance of the "to-good-to-treat" concept. Contrary to DEFUSE and EPITHET we chose not to screen patients via CT to keep door-to-MRI times shorter. The inclusion rate is significantly higher in 1000Plus than in both studies. See Table 2 for a comparative synopsis of 1000Plus, DEFUSE and EPITHET.

**Table 2:** Comparative synopsis of the 1000Plus, EPITHET and DEFUSE projects

	1000Plus	DEFUSE	EPITHET
*Time Window (onset-imaging)*	0-24 hours	0-6 hours receiving alteplase	3-6 hours receiving alteplase

*Imaging for Patient selection*	MRI	CT	CT

*Patients (to be) included*	1200	74	101

*PI post-processing*	MTT maps with and without delay correction	Tmax (time to peak without delay correction)	Tmax (time to peak without delay correction)

### MRI Protocol

Imaging at 3T enables to choose a higher resolution, shorter times of examination or a combination of both [18,19]. Using both approaches a high spatial resolution for DWI (voxel size 1.2 × 1.2 × 2.5 mm) and a good time resolution for perfusion imaging can improve the clinical utility of a stroke MRI protocol. An examination lasting less than 15 minutes enables to detect infarction, exclude hemorrhage, show chronic lesions, and characterize the vessel status and the size of a perfusion deficit. Having a purely research dedicated 3T scanner next door to our stroke unit is a unique condition for clinical stroke research. This vicinity helps us to design longitudinal studies with short intervals between MRI scans. As an example patients are included into a substudy to document the time of lesion conversion on FLAIR images with 5 examinations on day of admission. Furthermore we are evaluating data sets of patients who underwent TOF as well as contrast agent high resolution MR angiography.

The exclusion of ICH is crucial to perform thrombolytic therapy. There is still a traditional resistance against using MRI to detect haemorrhages, but MRI and CT are equivalently validated instruments for this demand in stroke patients [2,3]. Although haemorrhage can easily be identified on the perfusion imaging source data we decided to perform a conventional T2* weighted sequence due to advantages in spatial resolution and a limited extend of skull base artefacts at 3T.

Diffusion weighted imaging (DWI) is widely accepted as sensitive to the actual size of infarction *at the moment *of examination, while it underestimates the final size [20]. Recently evidence emerged, that approximately 1/3 of DWI lesions are normalizing to a certain degree if they were diagnosed in a 3 h time window [21,22]. This reversibility seems to depend on delay of imaging, reperfusion and localization of the infarct [23]. However, according to our experience, lesion regression is rare beyond a 3 hour time window. We study all recent timeframes after ictus to get a representative picture of DWI lesion evolution in acute stroke. Although it has never been studied in detail we add a day 5-7 control to validate whether reversibility was permanent or transient.

Several studies tried to evaluate the best possible modality to raise sensitivity for final infarct size by adding perfusion imaging to the diagnostic protocol [20,24,25]. However, the ideal technique of PI and especially its post-processing has not yet been established [20,24]. Several publications found differing results about PI lesion prediction depending on different processing methods [24]. Yamada and co-workers found the highest sensitivity for MTT maps. They showed sensitivity for final infarct volume of 85-94% based on MTT, generated from data based on 12 patients with persistent vessel occlusion. This high sensitivity was associated with a positive predictive value of 54-61% [20]. In contrast, Parsons et al. found rCBF most accurately predicts infarct growth in mismatch patients [25]. EPITHET and DEFUSE both used non-delay-corrected Tmax approaches to determine perfusion deficit [14,15]. The potential limitation of a Tmax approach is its sensitivity to chronic perfusion deficits. Depending on the presence and diameter of communicating arteries of the circle of Willis perfusion compensation can vary. We decided to use MTT maps after sSVD (standard singular value decomposition) and bSVD (block-circulant SVD) deconvolution. Therefore, we will be able to compare a non-delay-corrected (sSVD) to a delay-corrected (bSVD) approach. Furthermore we add vessel occlusion as an additional parameter to evaluate the mismatch model. Neither DEFUSE nor EPITHET took the target in account for the question of superior prediction of final infarct size. Therefore, we integrated a time-of-flight (TOF) MR angiography into our protocol to assess vessel status. TOF-MRA has been used in several trials before [4,5,10,12]. It is a robust non-invasive tool to determine the vessel-status in acute stroke patients with a sensitivity of 84.2% and a specificity of 84.6% compared to conventional angiograms [26].

FLAIR was included to assess the final size of the infarction on day 5-7. Furthermore FLAIR helps to evaluate the age of the stroke and to diagnose white matter lesions. With those five sequences a maximum of necessary information can be obtained with minimal time consumption.

### Possible Consequences

The aim of this study is to describe the incidence of mismatch and the predictive value of PI for final lesion size and functional outcome depending on door-to-imaging time and vascular recanalization. It is crucial to standardize PI for future randomized clinical trials as for individual therapeutic decisions. We expect to make a significant contribution to this challenging task with "1000Plus".

## Abbreviations

AIF: arterial input function; ASIST: Acute Stroke Imaging Standardization Group; bSVD: block-circulant SVD; DEDAS: Dose Escalation of Desmoteplase for Acute Ischemic Stroke; DEFUSE: Diffusion and Perfusion Imaging Evaluation For Understanding Stroke Evolution; DIAS: Desmoteplase In Acute Ischemic Stroke; DWI: diffusion-weighted imaging; EPITHET: Echoplanar Imaging Thrombolytic Evaluation Trial; FLAIR: Fluid-attenuated inverse recovery; ICH: intracerebral hemorrhage; MCA: middle cerebral artery; MRA: MR angiographies; mRS: modified Rankin Scale; MTT: mean transit time; NIHSS: National Institute of Health Stroke Scale; PI: perfusion imaging; PMA: Perfusion Mismatch Analyzer; ROI: region of interest; sSVD: standard singular value decomposition; TOF MRA: time-of-flight MR-Angiography

## Competing interests

PD. JBF reports receiving consulting, lecture and advisory board fees by BMS, Siemens, Philips, Perceptive, BioImaging Technologies, Boehringer Ingelheim, Lundbeck and Sygnis. GJJ reports lecture and consulting fees by Boehringer Ingelheim, BMS, Sanofi, Genzyme and was granted a fund by UCB. ME reports receiving consulting, lecture, grant and/or advisory board fees by BMS, Sanofi, Boehringer Ingelheim, Novartis, Pfizer and AstraZeneca.

## Authors' contributions

JBF, GJJ, AV and MEn initiated the study. JBF wrote the protocol. CX, PB and BH provide KK with feedback to optimize PMA software for large patient cohorts. BH, SP, MEb, GO, MR and WUS will do the patient inclusion and MRI examinations under supervision of JBF. KJ is the study coordinator. PM will do the statistical calculations. All authors have read and approved the final manuscript.

## Pre-publication history

The pre-publication history for this paper can be accessed here:

http://www.biomedcentral.com/1471-2377/9/60/prepub
